# Furthering the Capabilities of Diffusive-Gradient Passive Samplers for Per- and Polyfluoroalkyl Substances

**DOI:** 10.1021/acs.est.4c14136

**Published:** 2025-05-08

**Authors:** Jarod Snook, Jitka Becanova, Simon Vojta, Rainer Lohmann

**Affiliations:** † 54083 University of Rhode Island Graduate School of Oceanography, 215 S Ferry Rd, Narragansett, Rhode Island 02882, United States

**Keywords:** PFAS, passive sampling, water quality, DGT, validation, monitoring, field deployment

## Abstract

Per- and polyfluoroalkyl substances (PFAS) are chemical pollutants of growing concern for many stakeholders. Due to their ubiquity, persistence in the environment, and potential for toxicity at low environmental concentrations, it is necessary to have convenient and reliable methods to measure PFAS in natural waters. Passive sampling methods (in situ preconcentration of PFAS) may be suitable for monitoring situations. One passive sampling design successfully employed for other, well studied contaminants (e.g., methylmercury) is the diffusive gradient in thin film sampler (DGT). However, the application of DGT for PFAS requires development and validation. Here, we iterate on previous PFAS-DGT studies by introducing a redesigned diffusive gradient sampler for PFAS in water and show that it reliably measures 25 PFAS in water, consistent with diffusion theory. Diffusion and whole-sampler uptake rates consistently agreed with model predictions within ±50% relative difference, including when tested at cold temperature (5 °C). In field and laboratory deployments, DGT samplers measured PFAS concentrations within ±23% of grab sample results on average in each case—better performance than codeployed microporous polyethylene tube passive samplers. Based on the evidence in this study, the DGT passive sampler is a promising tool for consistently and accurately passively sampling PFAS in natural waters.

## Graphical abstract

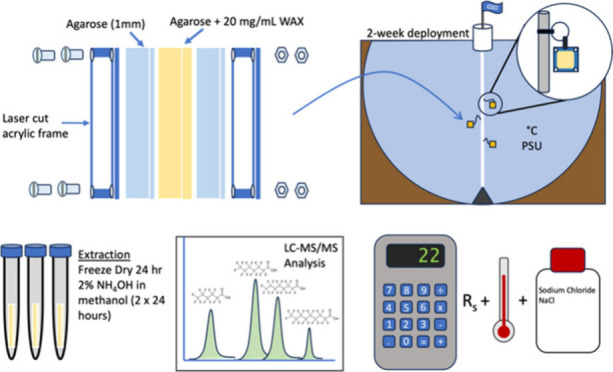

## Introduction

Per- and polyfluoroalkyl substances (PFAS) are a class of synthetic chemicals that are a growing concern for governmental regulators, water management entities, and the public. Due to their desirable waterproofing, stain repellant, and firefighting properties, PFAS manufacturing has increased over time, expanded to many industries, and the class has grown to several thousand individual compounds containing the moiety C_*n*_F_2*n*+1_.^1^ PFAS owe both their industrial usefulness and harmful environmental and biological longevity to the strong carbon–fluorine bonds characteristic of their chemical class. The long lifetime and relative mobility of PFAS in the environment causes them to be detected in various matrices, such as water, soil and sediment, air, dust, and biological organisms including humans.^2–6^ PFAS are also potent toxicants, with potential for long-term toxic effects at very low (part per trillion) exposure concentrations.^7^ Two PFAS, Perfluorooctanoic acid (PFOA) and perfluorooctane sulfonic acid (PFOS), were recently classified as known and possible carcinogens to humans, respectively.^8^

It is imperative for addressing PFAS contamination to have reliable and convenient measurement tools. Methods for detecting PFAS, especially in drinking and surface water, must be (1) accurate, as remediation goals depend on measured concentrations; (2) convenient, due to the large quantity and sizes of contaminated zones; and (3) affordable. Currently, most assessments of surface water PFAS contamination use solid-phase extraction of water grab-samples (following EPA Method 1633).^9^ Though precise, this active-sampling method has limitations—it is labor intensive, can require large (>1L) water samples for low detection limits, has high shipping costs, and provides a limited temporal snapshot of PFAS concentrations. Passive sampling methods, where a device preconcentrates PFAS *in situ*, have the potential to overcome these limitations, though considerable effort is still needed to validate them for PFAS in complex water matrices.^10,11^

Several passive sampling devices have been tested, calibrated, and used for measuring PFAS in natural waters. Designs such as the microporous polyethylene tube,^12,13^ high density polyethylene-housed Cu­(II)-PEI-SOMS adsorbent,^14^ PFAS INSIGHT,^15^ diffusion cell,^16^ graphene hydrogel,^17^ or POCIS^18^ passive samplers for PFAS have successfully measured PFAS in natural waters via equilibrium or kinetic mechanisms. However, these sampling devices may be hindered by various drawbacks such as effects of environmental variables (flow rate, pH, etc.), complex models for determining sampling rate or K_d_, or limited applicability to different water matrices (surface water vs groundwater). Thus, novel passive sampler designs should be accurate (within a factor of 2), time-integrative, and easy to use/report dissolved concentrations under a range of possible field conditions.

The diffusive gradient in thin-film (DGT) passive sampler type has been successfully used for several legacy contaminant types (e.g., trace metals, pharmaceuticals, pesticides, etc.).^19–21^ Contaminants validated for DGT sampling encompass a wide range of chemical properties, including organic/inorganic, polar/apolar, and a wide range of molecular weights.^22,23^ For contaminant specificity and performance optimization, a wide variety of DGT configurations have been developed. Construction materials are most often polyacrylamide or agarose hydrogels, often with a protective outer membrane made of nitrocellulose, glass microfiber, PTFE, PES, or other materials. Adsorbent resins used in DGT passive sampler designs vary widely even within one contaminant type.^20^ The unique polarity and surfactant properties of PFAS require extensive validation steps (including verification of the underlying DGT theory) prior to DGT use for quantifying environmental PFAS. DGT validation for PFAS is underway, with calibration and successful field validation for up to 16 PFAS in wastewater treatment plants, freshwater, and brackish matrices.^24–27^ With the large number of known PFAS compounds and increasing attention on PFAS contamination from regulators and the public, expansion of the validated analyte list for DGT, increased accuracy and precision in measurement, and expansion to additional natural water types are warranted. We also aim to optimize the passive sampler design to avoid potential PFAS-specific interferences such as omitting an outer membrane and selecting an adsorbent designed for PFAS retention.

DGT passive samplers operate by placing a diffusive hydrogel layer (typically 0.8–1 mm) between a binding layer (specific for target compounds) and the bulk water body.^20^ The thickness of the diffusive layer provides the rate limiting step and thus minimizes effects of a changing water boundary layer in variable flow conditions. This allows for consistent sampling rates, *R*_s_, for a given target compound—one of the strengths of the sampler design. Under idealized conditions, the uptake of contaminants from water is controlled only by the diffusion (coefficient) of the contaminant through the diffusive layer material. For most organic contaminant sampling, agarose hydrogel is favored over polyacrylamide for providing higher sampling rates, though it is more fragile. However, deviations from standard temperature will affect diffusion rates, as could changes in the salinity, DOC, or other environmental properties. Salinity, in particular, has been shown to affect PFAS strongly in adsorption studies,^28,29^ and to affect DGT sampling for other contaminants.^20,30^

Here, we demonstrate a redesigned symmetrical DGT sampler for PFAS which takes up compounds on both sides for increased sampling rate and PFAS specific adsorbent in the binding layer. The sampler also lacks an outer membrane to prevent the adsorption of PFAS on the outside of the sampler. We assessed its sampling rate for 25 PFAS (the sum of branched isomers were considered separately from linear isomers) via two methods: by determining diffusion coefficients and associated theoretical sampling rates and in-practice sampling rates via laboratory deployments of whole samplers. Additionally, we assess environmental factors that affect its uptake and confirm sampling rates in multiple complex water matrices via field deployments.

## Materials and Methods

### DGT Sampler Construction

The DGT samplers were constructed primarily of 1.5% (w/v) agarose gel in high-purity LC-MS water (Fisher Scientific, New Hampshire, USA). Agarose powder (Sigma-Aldrich, Missouri, USA) was added to water in the above ratio and heated and stirred until dissolved into aqueous solution (90 °C). The solution was poured between glass plates separated by a 1 mm silicone spacer and held together by clamps. The gel was allowed to cool and solidify before glass plate disassembly. Agarose hydrogels for DGT binding layers were produced similarly, with the addition of 20 mg mL^−1^ precleaned Oasis WAX adsorbent dispersed in the agarose solution, which was poured between glass plates with 2 mm spacing. Oasis WAX adsorbent was selected due to its high affinity and specificity for PFAS adsorbates.^31^

Samplers were assembled by layering binding and diffusive layers between 3.75 × 3.75 × 0.159 cm laser-cut acrylic plastic frames (as in Figure 1). The assembly was secured with a nylon plastic nut/bolt at each corner, and the side edges of the sampler were covered and sealed with a strip of Parafilm. All sampler materials were tested for PFAS by methanol extraction prior to selection as construction materials and showed no detectable PFAS. The combined sampling area (both faces) was 21.13 cm^2^.

**Figure 1:**
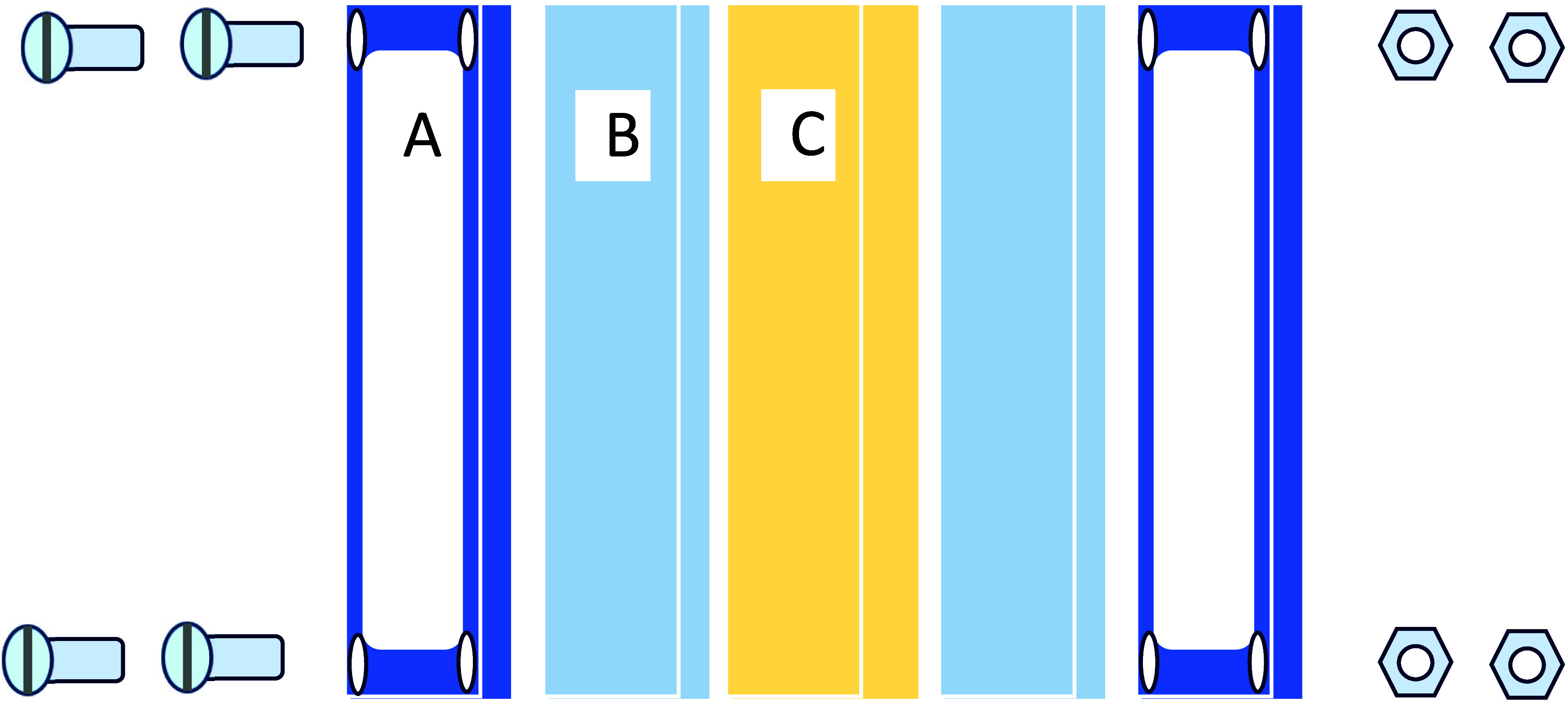
Diagram of two-sided DGT sampler design with acrylic plastic frames (A), agarose diffusive layers (B), and 20 mg/mL WAX adsorbent dispersed in an agarose gel binding layer (C). The overall dimensions of the sampler were 3.75 × 3.75 × 0.718 cm when assembled (not including nylon nut/bolt fasteners). The acrylic frames also included a small slot on the face of the frame allowing for deployment via zip-tie without compromising the sampling window (not shown). See Image S1 for a photograph of the assembled DGT sampler.

### Slice Stacking *D*_H_ Determination

Agarose diffusion experiments were conducted via a hydrogel slice-stacking method.^32,33^ For each experiment, seven agarose sheets (3.75 × 3.75 × 0.1 cm) were prepared identically to sampler diffusive layers. One sheet was immersed in a PFAS mixture in deionized water solution and shaken at 100 rpm until equilibrium (at least 72 h) resulting in nominal concentrations of ∼2 ng/mL for each PFAS (see Table S1 for details on PFAS stocks used in spiking solutions). The room-temperature prespiked and clean agarose sheets were assembled in a neat stack with the spiked sheet at the bottom of the stack. The entire stack was sealed within a plastic bag and placed in a 25 °C temperature chamber for 90 min (see Image S2). After the specified time, the stack was disassembled and individual sheets were stored in 15 mL polyethylene conical tubes. Each sheet was spiked with mass-labeled PFAS internal standards and extracted via 2% ammonium hydroxide in methanol solution (2x 24 h extractions at 100 rpm with 5 mL solvent each). Agarose extracts were evaporated to a 0.5 mL volume under a gentle stream of nitrogen in a 37 °C dry bath before instrumental analysis.

Concentration of each PFAS in each agarose layer was plotted as a function of position (in mm, with the centroid of each slice as reference, i.e., spiked slice at 0 mm, second slice at 1 mm, etc.). Using Python nonlinear least-squares fitting, the distribution of PFAS was fit with eq 1,^34^ providing the agarose hydrogel diffusion coefficient of each. In eq 1, *x* represents diffusion distance and *t* diffusion time. *D* (diffusion coefficient) and *M* (total PFAS mass) were predicted via curve fitting. At least four data points (>0) were deemed adequate to generate an accurate curve fit and *D*_H_ value. Diffusion results for PFAS compounds that did not meet this criterion were not reported. Effective diffusion coefficients in agarose (*D*_e_) were also predicted via the average of diffusion based on Archie’s Law (eq 2) and two additional models^35^ (Supplemental Text 1) for comparison with experimental data.^26,36^ Coefficients in eq 2 for agarose are ε (porosity) = 0.98 and *m* = 2. *M*_w_ is the molecular weight, and *D*_H_ refers to diffusion in porous media, such as an agarose hydrogel. See Zhang and Davison and Challis et al. for more details on eq 2.^26,37^

1 $$C=\frac{M}{{\left(\pi D_{H}t\right)}^{1/2}}\times e^{\left(\frac{-x^{2}}{4D_{H}t}\right)}$$

2 $$D_{H}=\frac{3.3\times 10^{-5}\varepsilon^{m}}{\sqrt[{3}]{M_{w}}}$$

Furthermore, modeled or experimental diffusion coefficients may be adjusted for varying temperature via eq 3.^11,23^ To assess the validity of the temperature adjustment model, we performed the slice-stacking diffusion experiment in a 5 °C temperature chamber (with agarose gels acclimated to the cool temperature for 2 h prior to starting the stack experiment). PFAS diffusion coefficients in agarose measured in the 5 °C experiment were compared to the average modeled diffusion coefficients adjusted to 5 °C via eq 3.

3 $$\log D_{T}=\frac{1.37023\left(T-25\right)+0.000836{\left(T-25\right)}^{2}}{109+T}+\log\frac{D_{298\;K}\left(273+T\right)}{298}$$

### Laboratory Flow-Tank Calibration

Experimental sampling rates were determined via whole-sampler deployments in a 100 L polyethylene flow tank. Temperature was maintained at 16 ± 2 °C and flow rate was ∼15 cm s^–1^ throughout the deployment period. PFAS compounds were spiked into the flow tank via the standards listed in Table S1. PFAS concentrations in the flow tank ranged from 3.49–79.8 ng L^–1^ depending on the compound (though concentration for each PFAS remained consistent throughout the experiment). Duplicate samplers were removed from the flow tank at 4, 12, and 26 h, and triplicate samplers removed at 2, 7, 15, and 21 days. The 50 mL water grab-samples were taken at deployment and each removal. PFAS *M*_s_/*C*_w_ (as shown in eq 4, with *M*_s_ being PFAS mass on the binding layer of the sampler and *C*_w_ grab-sample measured concentration of PFAS in water) for each compound were plotted over the deployment length then fit with linear least-squares regression to determine sampling rate and confirm linear uptake over the 21 day deployment period.

4 $$C_{w}=\frac{M_{s}}{R_{s}\times t}$$

In a separate experiment, the sampling rates for each PFAS determined above were used to measure the flow-tank water concentration after a 14 day deployment. Conditions in the tank were similar to the calibration experiment, but water grab samples (taken at deployment and recovery) were compared with sampler-measured water concentration (eq 4) as a “challenge” experiment for the previously determined sampling rates.

### Salinity Effects Experiments

Previous laboratory experiments (unpublished) have suggested a strong salinity effect on the activity of PFAS and their uptake into passive samplers. Thus, to investigate PFAS salting-out or other salinity effects on DGT passive samplers in a more realistic deployment, a seawater mesocosm-based DGT deployment was conducted. Duplicate DGT passive samplers were deployed in 300 L tanks containing constant, slow-flowing, sand-filtered seawater from Narragansett Bay. At the deployment and recovery of samplers 1 L water grab samples were taken and analyzed for PFAS. Temperature was maintained at 18 ± 1 °C and salinity was 29.8–30 ppt throughout the deployment. The PFAS concentrations measured by DGT under saltwater conditions were compared to grab sample measurements.

### Field Deployments

Two field demonstrations were conducted that tested the DGT sampler capability against grab-sampled water and established passive samplers (microporous polyethylene tube sampler, MPT). Field conditions and deployment set-ups differed between field sampling campaigns and are described specifically for each below. In general, samplers were deployed via zip-tie on rope lines via either anchor and float or weighted rope attached to a surface structure (top-down method). Water grab samples and temperature measurements were taken as often as feasible; in each case here, separate water measurements were taken at least at deployment and recovery of passive samplers. Water grab samples and passive samplers were extracted and analyzed via LC-MS-q-ToF as described below. For comparison, DGT samplers were deployed alongside MPT. These were handled as described in Gardiner et al. and Dunn et al.^13,38^ Additional details on deployment, extraction, and water concentration calculation are provided in Supplemental Text 2. Modeled-diffusion-based sampling rates were used for DGT samplers to measure field water concentrations (these *R*_s_ were optimal, as described below), whereas MPT tubes utilized measured sampling rates from prior research^13,38,39^ to appropriately match water temperature and/or deployment length conditions.

### DGT Sampler Extraction

DGT samplers were extracted by first disassembling the sampler and placing the binding layer in a 15 mL polypropylene tube. The binding layer was then spiked with internal standard solution and freeze-dried for 24 h to remove all water content and optimize extraction efficiency. When completely dry, 5 mL of 2% ammonium hydroxide in LC-MS-grade methanol solution was added and allowed to extract whole, dry binding layers on a shaker table (100 rpm) for 24 h. Methanol extracts were poured into a fresh tube, and the extraction was repeated with a second 5 mL aliquot of ammonium hydroxide/methanol solution, combining both extracts upon completion. The combined methanol extract was then evaporated under a gentle stream of nitrogen to a final volume of 0.5 mL, spiked with isotopically labeled recovery standard (distinct from internal standards) PFAS solution, and prepared for LC-MS/MS analysis described below.

### Water Sample Solid Phase Extraction

Water phases were extracted using a modified EPA Method 1633.^9,17^ Solid phase extraction (SPE) using 150 mg Oasis WAX SPE cartridges was performed for 50–500 mL of water (depending on estimated concentration and expected instrumental detection limits). The water sample was spiked with isotopically labeled internal standard PFAS (used for extraction efficiency correction) and passed through SPE columns. The SPE columns were then eluted with 2% ammonium hydroxide in LC-MS methanol. Methanol extracts were concentrated with a gentle stream of nitrogen to a final volume of 0.5 mL. Water extracts from field samples were also spiked with mass-labeled recovery standard solution prior to preparation for instrumental analysis.

### Instrumental Analysis and QA/QC

First, 40 uL of methanol extracts from either extraction process were diluted by a factor of 2.5 into a final 100 uL solution of 40:60 LC-MS methanol:10 mM ammonium acetate in LC water. This solution was injected into a SCIEX ExionLC AC UHPLC system coupled to a SCIEX X500R quadrupole time-of-flight tandem mass spectrometer (QTOF MSMS), and results were analyzed in Sciex data processing software (see Supplemental Text 3 for additional LC-MS/MS details and Table S2 for full target compound list).

Method detection limits (Table S3) for each type of sample (water extract and DGT extract) for each PFAS were calculated via the average + 3x the standard deviation of process blank samples with detected PFAS. For PFAS with no detections in blank samples, the instrumental detection limit (signal-to-noise ratio = 3) was used. Field water samples provided by collaborators were reported with individual MDLs calculated via EPA Method 1633 recommendations. Samples below method detection limits were removed or reported as “<MDL” as appropriate. All blanks were below method detection limits for all target PFAS.

Additionally, laboratory DGT samplers were tested for extraction efficiency via native-PFAS spiked binding layers extracted alongside laboratory samples. A known quantity of a high-purity PFAS mixture (Wellington Laboratories, Inc., Ontario, CA) was added to the DGT binding layers, spiked with internal standards, and extracted as described above. Resulting concentration on the binding layer was compared with the known mass spiked (Table S4). The use of the analytical standard mixture in this experiment allowed for extraction efficiency calculations of 30 PFAS (including each detected in field-deployed DGT). All field-deployed DGT passive samplers also underwent an extraction efficiency check via comparison of internal standard recovery to a separate mass-labeled PFAS recovery standard (internal standard recoveries for all field samples are included in Table S5).

### Data Analysis

Sampler calibration experiments were quantified by plotting the sampler uptake of each PFAS for each time point in the calibration experiment. The slope of the linear fit (via Microsoft Excel LINEST function, with *y*-intercept set to 0) for each uptake curve was used to determine the overall DGT sampling rate for each PFAS, as per eq 4. For comparison, agarose gel diffusion coefficients determined through the slice-stacking method and via diffusion models were also converted into theoretical sampling rates using sampler dimensions and eq 5, where *A* is sampling window area and Δ*x* is diffusive layer thickness. For sampling rate calculations, three diffusion models were averaged to provide robust average *R*_s_ and uncertainty values.

5 $$R_{s}=\frac{D_{H}\times A}{\Delta x}$$

To derive water concentrations, model-based sampling rates were used due to the general agreement between modeled and experimental values and the ability to model *R*_s_ for any detected PFAS.

## Results and Discussion

### DGT Sampler Extraction Performance

Individual and average recoveries of native-PFAS spiked into DGT binding layers are given in the SI (Table S4). Average recovery ranged from 76–149% (PFDS and HFPO–DA, respectively). Standard deviation among replicates was <35% for each PFAS. Considering typical analytical uncertainties in PFAS analysis, the extraction performance for the DGT sampler and proposed extraction procedure (freeze-dry and 2% ammonium hydroxide in methanol extraction) were deemed acceptable.

### Diffusion in Agarose Hydrogel

Diffusion coefficients in agarose, *D*_H_ (cm^2^ s^–1^), and values for 17 PFAS derived from slice-stack diffusion experiments at 25 °C displayed a narrow range (both experimentally and via model), with most common PFAS near 5 × 10^–6^ cm^2^ s^–1^, likely a result of their similar properties, molecular weights, and molar volumes (Figure 2 and Table S6, all curve fits are shown in Figure S1). This small range may be helpful, as it should provide a narrow and consistent range of expected sampling rates for the DGT sampler. As expected, diffusion coefficients were smaller for larger molecular weight PFAS, especially within the tested range of different carbon-chain length PFCAs. When comparing experimental to modeled *D*_H_, experimental values aligned well with model expectations, with a maximum relative percent difference (defined as the percent ratio of the absolute difference between two values divided by their average, RPD) of 48% (PFNA). The model predicted *D*_H_ values especially well for some PFCAs (≤5% RPD difference for PFPeA and PFHxA), but somewhat overpredicted for PFBA (+23% RPD) and underpredicted for longer chain (−48% for PFNA) PFAS. PFSAs were more consistent between experiment and model *D*_H_, with all showing <22% RPD. Some of the larger disagreements between model and experimental *D*_H_ also showed highest experimental uncertainty (derived from curve fitting residuals), meaning curve fitting to stack experiment data was less precise in these cases. This can be explained by the experiment timing being optimized for C6–C10 PFAS, resulting in less ideal data for curve fitting smaller or larger *M*_w_ PFAS. Diffusion coefficients were lower by 0.67–2.36 × 10^–6^ cm^2^ s^–1^ (11–46%, PFHxA and PFNA, respectively) in this study compared to previously reported PFAS diffusion in agarose hydrogel for the seven PFAS measured in both studies.^40^

**Figure 2:**
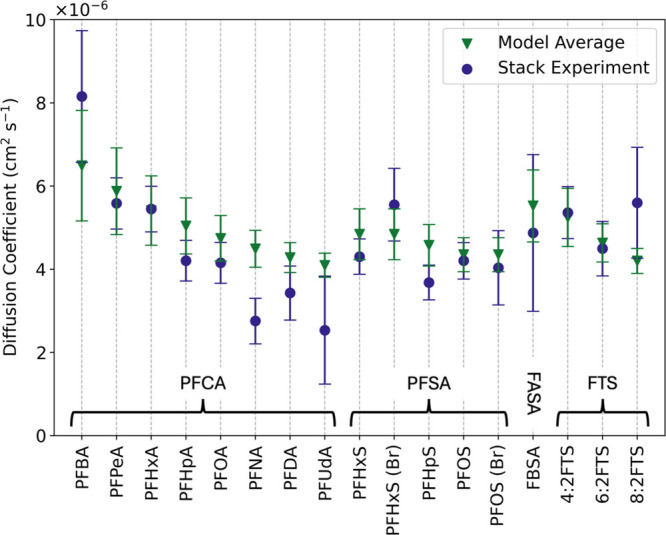
Comparison of model-based *D*_H_ prediction (average ± standard deviation of three models) with slice-stacking experiment results for 17 PFAS. Error bars on experimental results are the standard error resulting from curve-fit residuals.

### Temperature Effect on Diffusion

The slice-stacking diffusion coefficient method was also repeated in a climate control chamber held at 5 °C to determine the effect of temperature on diffusion through agarose and assess eq 3. Diffusion coefficients in agarose at 5 °C from both methods are displayed in Figure S2 and Table S6, which demonstrates the expected reduction in diffusion at lower temperature, and good agreement (<50% relative difference) between modeled and experimental diffusion values for eight PFAS with adequate data for curve fitting. Therefore, we consider eq 3 to represent an appropriate adjustment approach for PFAS diffusion in agarose (and, in turn, *R*_s_) over the environmental temperature range. Though these temperature results are as expected, it is important to consider the environmental temperature, resulting sampling rates, and expected concentrations of PFAS in the field when determining suitable passive sampler deployments times. If temperature fluctuation is expected, DGT samplers may need to be deployed with temperature loggers and more complex adjustments for time-averaged temperature used.

### Sampler Uptake

Sampler uptake was quantified by linear regression of the accumulated PFAS mass over time and described by the sampling rate (*R*_s_). Sampling rates for PFAS in laboratory flow-tank experiments are tabulated below (Table 1, with grab sample concentrations reported in Table S7). Sampling rates were consistent (and uptake linear) over the course of the 21 day experiment (e.g., Figure S3), and *R* ^2^ values are included in Table 1. For comparison, theoretical sampling rates converted from both experimental and modeled diffusion values (eq 2) are included as well. Uncertainty was propagated via (1) standard deviation of any replicates, (2) uncertainty of curve fits, and/or (3) standard deviation of several model predictions. Here, again, sampling rates are most consistent across methods for PFSAs and middle-chain-length PFCAs, with most disagreement for short PFCAs. For all PFCAs except HFPO–DA, and all PFSAs and FTSs, experimental *R*_s_ were within 55% relative difference (RPD) of model-predicted values, with 16 of 18 within 40% RPD. Sulfonamide PFAS (FASAs) and HFPO–DA performed somewhat worse, with 43–89% RPD between measured and model-predicted *R*_s_.

**Table 1:** Experimental and Model-Based Sampling Rates (mL day^–1^) for the DGT Passive Sampler at 16 °C^a^

compound_(*n*)_	experimental *R*_s_ (mL day^–1^)	experimental SE	*R* ^2^	model *R*_s_ (mL day^–1^)	model SD	RPD
PFBA_(24)_	41.1	1.0	0.987	62.3	12.7	41
PFPeA_(23)_	49.4	1.2	0.987	56.4	10.0	13
PFHxA_(24)_	60.1	1.6	0.984	51.9	8.0	15
PFHpA_(24)_	56.2	1.7	0.979	48.4	6.5	15
PFOA_(24)_	64.2	1.8	0.983	45.5	5.2	34
PFNA_(24)_	63.3	2.2	0.974	43.1	4.3	38
PFDA_(24)_	60.0	2.3	0.968	41.1	3.5	37
PFUdA_(23)_	51.1	2.1	0.964	39.3	2.8	26
PFDoA_(22)_	44.6	1.8	0.964	37.7	2.2	17
PFTrDA_(21)_	33.9	1.7	0.945	36.4	1.7	7
PFTeDA_(12)_	32.7	2.0	0.921	35.1	1.3	7
PFBS_(22)_	61.6	1.6	0.984	53.3	9.0	14
L-PFHxS_(23)_	70.2	2.2	0.979	46.4	5.9	35
Br-PFHxS_(22)_	81.5	2.6	0.977	46.4	5.9	55
PFHpS_(24)_	70.2	2.7	0.968	43.9	4.8	41
L-PFOS_(23)_	62.2	2.4	0.966	41.7	3.9	35
Br-PFOS_(23)_	72.9	3.1	0.961	41.7	3.9	54
FBSA_(22)_	26.8	0.8	0.981	53.0	8.3	43
FPeSA_(12)_	20.4	0.9	0.951	49.2	6.7	89
FHxSA_(22)_	20.6	0.7	0.974	46.2	5.4	77
FOSA_(24)_	20.1	0.7	0.970	41.6	3.6	70
HFPO–DA_(12)_	103.2	4.7	0.954	50.6	7.3	68
4:2 FTS_(22)_	46.1	1.2	0.984	50.3	6.7	9
6:2 FTS_(22)_	51.5	1.4	0.983	44.4	4.4	15
8:2 FTS_(22)_	46.4	1.9	0.965	40.3	2.9	14

a Experimental rates were determined by linear correlation of sampler uptake over time (*n* = number of detections used in linear fitting), with standard error (SE) propagated from the error on the fitted slope. *R* ^2^ values from the linear fit are also provided, as well as the relative percent difference (RPD) between experimental and modeled *R*_s_. Modeled values were generated by averaging three models for diffusion through the hydrogel, adjusting *D*_H_ to 16 *°*C, and converting to *R*_s_. The standard deviation was propagated from the standard deviation (SD) of the model average. (Br-) refers to the sum of branched isomers of PFHxS or PFOS.

With the sum of evidence shown above, model-based sampling rates and temperature correction were selected for determining the PFAS water concentration via DGT passive sampling in the field. The average of three diffusion models used here showed generally good agreement with experimentally determined diffusion and sampling rates and can be simply calculated and extrapolated to a wide range of PFAS beyond those tested in flow-tank uptake experiments with a reasonable degree of certainty. The comparison of model-based and experimental *R*_s_ provides, in general, estimations within the factor of 2 sought for high-accuracy passive sampling, though with additional experimentation needed on FASA-type PFAS. Using the model-based method also ensures calculated sampling rates are supported by DGT sampling theory. For this reason, we chose to assess the sampler performance in further laboratory and field studies with model-based sampling rates and temperature correction only. We hypothesize that these methods will adequately predict PFAS water concentration in most freshwater environments. However, the selection of model-based sampling rates also introduces the potential for poorer results where model and experimental results disagreed (e.g., FASA-type PFAS). Thus, concentrations determined for these PFAS should be used with caution until further study can determine the cause and extent of model/experimental disagreement. For FASA compounds in particular, the use of a nonionic adsorbent in DGT samplers may improve performance. Field performance of FASA-type PFAS will be monitored closely to determine whether different *R*_s_ methodology is necessary.

Using a repeat of the flow-tank deployment, sampler performance was examined by predicting PFAS water concentration using modeled sampling rates reported above. The sampler-calculated water concentration was within 50% RPD of grab water samples for 19 of 23 PFAS (PFUnDA, PFDoDA, FBSA, and 8:2 FTS being >50% RPD; FPeSA and HFPO–DA had poor grab sample extraction recovery and were omitted) (Figure 3, Table S8). By linear least-squares fitting, we determined the linear correlation between sampler and grab sample measured concentrations. The linear fit had a slope not significantly different from 1 (good agreement) and a high *R* ^2^ (0.898). FBSA (indicated on Figure 3) was the only PFAS not within a factor of 2 of grab sample measurements. Other than FASA-type PFAS, the calibration and challenge experiments demonstrate the sampler’s consistency of PFAS uptake and predictive ability in controlled laboratory conditions.

**Figure 3:**
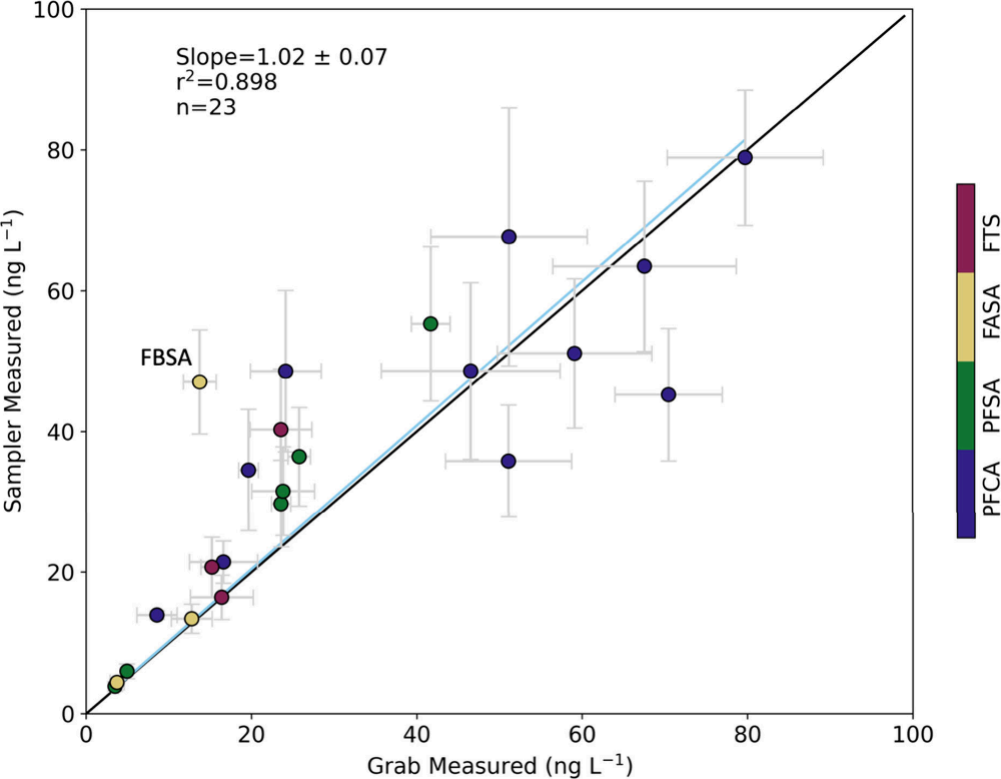
Sampler-predicted and measured water concentration in 100 L flow tank “challenge” experiment. The black line represents ideal 1:1 agreement between the passive sampler (*n* = 2) and grab sample (*n* = 2) measured concentrations, and the blue line is the least-squares linear fit of the data. Uncertainty is the standard deviation of replicates for water grab samples and the propagated uncertainties of replicates and model-based uncertainty for DGT samplers.

### Salinity Effects on Uptake

Ten PFAS were detected by DGT passive samplers (11 were detected in grab samples) in saltwater mesocosm deployments; all were below 1 ng L^–1^. Unexpectedly, DGT-measured concentrations agreed well (within 51% RPD) with grab sample measurements using a temperature correction (to 18 °C) only (Table 2). No salinity correction was needed to adjust concentrations for ∼30 ppt salinity in this study. This result differed from prior unpublished batch experiments. Possible explanations for the differences in uptake between experiment types may be the more realistic deployment conditions in the mesocosm set up. Regardless, in the realistic deployment scenario tested here, DGT samplers measured PFAS concentrations well despite high salinity. For high-salinity deployments, shorter deployment times may still be recommended, as the agarose hydrogel construction was noticeably more fragile after long exposure to saltwater.

**Table 2:** Water Concentrations (ng L^–1^) Measured by Each DGT Sampler and by Duplicate Grab Samples in a Saltwater Mesocosm Deployment^a^

compound	DGT 1 (ng L^–1^)	DGT 2 (ng L^–1^)	grab sample (ng L^–1^)
PFPeA	0.29 ± 0.05	0.30 ± 0.05	0.49 ± 0.14
PFHxA	0.35 ± 0.05	0.32 ± 0.05	0.43 ± 0.14
PFHpA	0.30 ± 0.04	0.31 ± 0.04	0.20 ± 0.03
PFOA	0.33 ± 0.04	0.33 ± 0.04	0.30 ± 0.04
PFNA	ND	ND	0.04 ± 0.001
PFDA	0.04 ± 0.003	ND	0.03 ± 0.001
PFBS	0.29 ± 0.05	0.23 ± 0.04	0.34 ± 0.09
L-PFHxS	0.13 ± 0.02	0.13 ± 0.02	0.13 ± 0.02
Br-PFHxS	0.03 ± 0.004	0.03 ± 0.003	0.05 ± 0.02
L-PFOS	0.10 ± 0.01	0.07 ± 0.006	0.09 ± 0.04
Br-PFOS	0.06 ± 0.005	0.06 ± 0.006	0.07 ± 0.02

a Grab sample measured concentrations are the average of both grab samples, and the uncertainty represents the higher and lower measured concentration.

Recently, poor mass binding was seen in estuarine DGT deployments for PFAS in a recent study.^27^ The authors suggested competition from other ions resulted in poor mass binding of PFAS in estuary and sea deployments. Our results do not show this effect at lower PFAS concentration (<1 vs 10s–100s ng L^–1^) and higher salinity (30 vs 14 ppt), though source water in our study was sand-filtered. Nor did our results indicate an increase in PFAS aqueous activity (and thus diffusion-based uptake) with ionic strength, as suggested by previous partitioning studies^29,41^ and unpublished batch experiments. Salinity did not affect measured concentrations in mesocosm deployments, potentially due to the very low dissolved PFAS concentrations in the source water. Under conditions similar to those for our mesocosm deployment, no salinity correction is needed for PFAS-DGT passive sampling. The lack of effect demonstrated here will be monitored in future field deployments, and the differences between previous experiments and our results should be the subject of additional mechanistic studies.

### Field Demonstration

Passive sampler deployments under various field environmental conditions showed the good performance of the DGT-sampler relative to water grab samples. In field demonstration 1, nine DGT samplers were deployed in lakes, a pond, and stream near Duluth, MN (encompassing unmeasured but distinct flow conditions). A total of four corresponding water grab samples were taken every other week over a 42 day deployment period. Samplers were discolored from the water matrix but otherwise remained intact upon recovery. Results from the passive samplers were compared with grab sample PFAS measurements by an outside laboratory on behalf of U.S. EPA (Figure 4 with the 1:1 line being perfect alignment between both methods, Table S9) in a similar manner to the laboratory experiment above. Passive sampler predicted water concentrations were calculated by the averaged diffusion coefficient models converted to sampling rates using eq 5.

**Figure 4:**
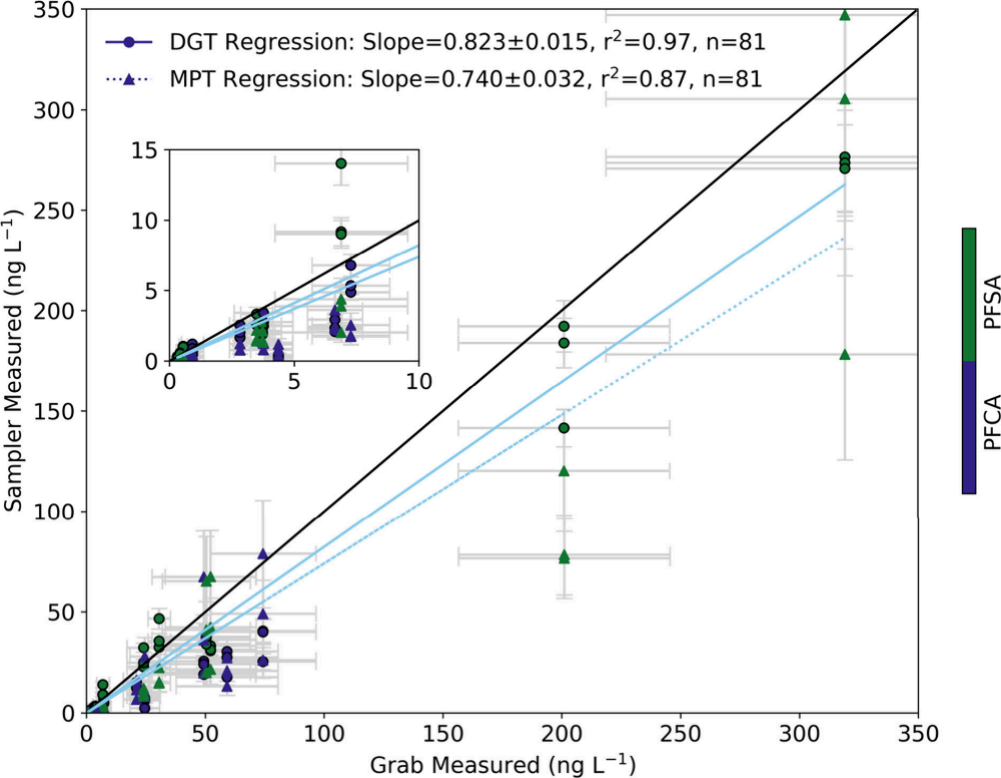
Comparison of DGT (circles) and MPT (triangles) passive sampler calculated water concentration and average water grab sample concentration for all detected PFAS and all sites in the Duluth, MN region deployments. Horizontal error bars represent the standard deviation of the measured water concentration. Inset is a magnified depiction of the low-concentration detections. Vertical error bars reflect 1 st.dev. of the average modeled *R*_s_ (*n* = 3)

Both passive samplers show good performance when compared with grab sample results overall, with DGT samplers matching more closely (slope = 0.82 vs 0.74 for MPTs). The ratio between passive and grab sample measured concentration for each detection is also depicted in Figure S4. Averaging each of these ratios results in a ratio of 0.77 for DGT:grabs (0.49 for MPT:grabs), i.e., the DGT concentrations were 77% of grab samples when each detection is averaged equally (as opposed to linear fits above, which can be influenced by high concentration points more strongly). In total, 73 of 81 PFAS detections with DGT were within ±100% RPD of grab sample values. Of those outside this threshold, six of eight were PFBA (all PFBA detections in the campaign), indicating poor performance with PFBA in general. It is possible that PFBA, which has known analytical interferences, was measured at incorrectly high concentration in water grabs as these were the only samples measured in a separate laboratory in this study. In field demonstration 1, one sampler exceeded the PFAS mass load of our 21 day calibration sampler (1634 ng sum of target PFAS vs 1347 in the calibration). Because this sampler showed a similar performance compared to grabs as all others in the campaign, it was probably still operating in the linear uptake regime necessary for accurate DGT performance and included in the study. We thus conclude the DGT capacity to be at least 1634 ng of total PFAS while remaining in the linear uptake regime.

For comparison, MPT passive samplers disagreed with grab samples by more than ±100% RPD in 20/81 PFAS detections, encompassing a larger diversity of PFAS compounds. A potential source of this uncertainty in MPT-derived concentrations is the lower extraction efficiency, as measured by internal standard recoveries (Table S5). The MPT had somewhat better performance with PFBA (three of six detections were <100% RPD), but also generally measured lower PFBA concentration than water grab samples, similar to DGT (Figure S4).

In field demonstration 2, four DGT and MPT samplers were deployed for 30–31 days in 2.5 °C (average of deployment and recovery measurement) freshwater stream known to be contaminated with PFAS, with three recovered intact (one damaged sampler was omitted from further analysis) at the end of the deployment period. Water grab-samples and temperature measurements were taken at deployment and recovery. Here, passive sampler predicted water concentrations were calculated by adjusting the diffusion coefficient model average to 2.5 °C via eq 3 and converting to sampling rates using eq 5 (Figure 5, Table S10). Both passive samplers again systematically measured lower water concentration than grabs, with DGT sampler vs grab sample linear fit slope = 0.73 and MPT slope = 0.57. However, all PFAS detections for DGT were within ±100% RPD compared to grab samples in this deployment, with 6/36 outside this threshold for MPT (though, each of these discrepancies were for detections <1 ng L^–1^). Ratios between passive and grab sample concentrations for each detection can again be seen in Figure S5. Overall, the average ratio in field deployment 2 was 0.89 for DGT and 0.69 to MPT. Compared to field demonstration 1, the DGT passive sampler underpredicted high-concentration PFAS more (comparison of DGT slopes in Figure 4 and Figure 5) but performed better overall (comparison of average ratio). This may be a result of the shorter deployment and relatively lower PFAS concentrations found there. The otherwise similar performance between field demonstrations 1 and 2 (an assortment of streams, ponds, and lakes) also provides further evidence for the DGT sampler’s resistance to variable flow conditions, an advantage over other PFAS passive sampler types. From both field demonstrations, the DGT samplers provided more accurate measures of the dissolved PFAS concentration in water than those derived from the MPT passive samplers, especially in cold water conditions.

**Figure 5:**
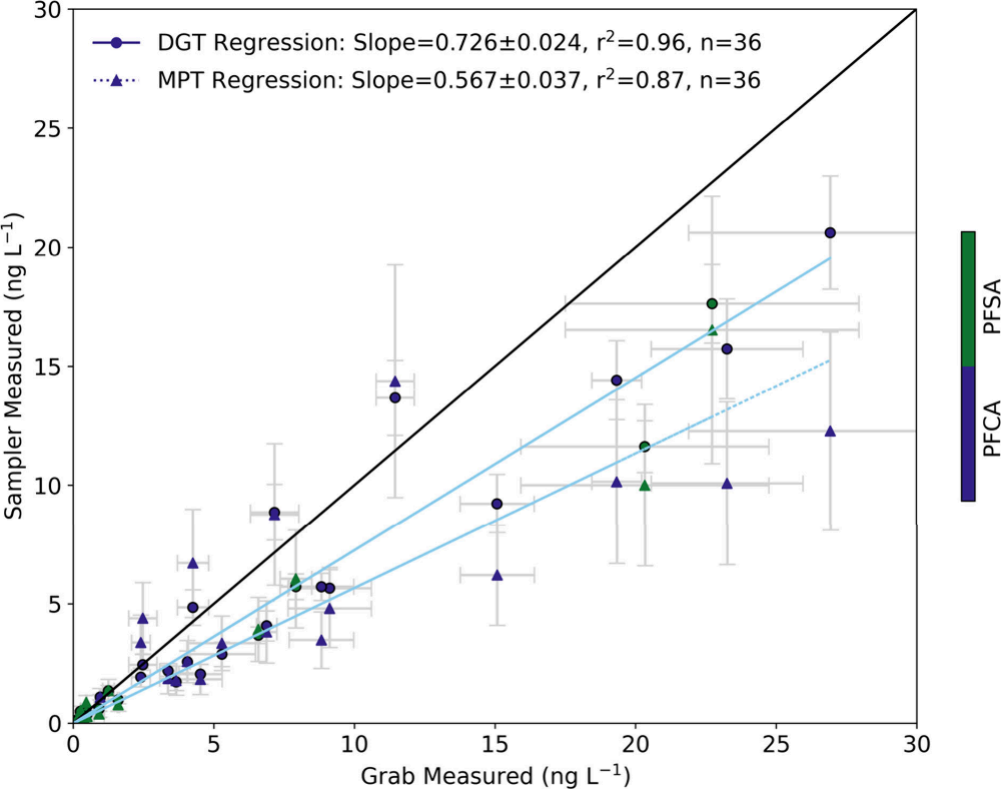
Comparison of DGT (circles) and MPT (triangles) passive sampler calculated water concentration and average water grab sample concentration for all detected PFAS and all sites in the Maine deployments. Horizontal error bars represent minimum and maximum water concentrations measured in water grab samples. Differences in sampler performance (i.e., deviations from solid black 1:1 line) are expected due to fluctuations in environmental PFAS concentration.

Both field demonstrations showed consistent underprediction of water concentration compared with grab samples. Typical sources of uncertainty between field deployed passive samplers and grab-sampling include fluctuations in actual contaminant concentration, differences in freely dissolved and whole-water contaminant concentration, and analytical uncertainties.^42–44^ Here, the consistency of the underprediction between not only two deployment campaigns but also two passive samplers suggests the freely dissolved average water concentration to be below results derived from the two grab samples. Another potential source for this discrepancy may be diurnal changes in water temperature affecting PFAS interactions with particles and sediments in the water body, with less freely dissolved PFAS during colder parts of the day. However, these temperature fluctuations could also cause slower uptake into passive samplers—neither interaction being adequately captured by daytime-only deployment and recovery grab sample collection and temperature measurement. The particle-bound fraction of PFAS likely contributes some of the difference between passive sampler and grab sample measurements, regardless of whether diurnal fluctuations affect the quantity. Furthermore, it is possible the interactions causing the discrepancy between samplers and grabs were less present at one site in field demonstration 2—the group of sampler-measured concentrations above the 1:1 line are from the same site. This site-specific exception to the consistent underprediction emphasizes that the nature of grab sampling can also cause variable results. Regardless, the differences between DGT and grab sample PFAS measurement here are similar to or smaller than those typically reported in other PFAS passive sampling studies.^15,27,38,45^ In consideration of the multiple potential sources of uncertainties, the small difference between the PFAS concentration derived from grab and DGT-passive sampling is promising for the performance of the DGT sampler under variable field conditions.

### Implications

This study iterates on previous PFAS DGT passive sampling work^24–27^ by (1) expanding the analyte list from 16 (combined previous studies) to 25 types of PFAS and (2) introducing a successful modeling framework (modeling diffusion rates via multiple models, and converting to *R*_s_ via eq 5) to estimate sampling rate for additional untested PFAS based on molecular weight and molar volume. This method can be used for any PFAS with a known chemical structure to easily estimate the *R*_s_. We further demonstrate the usability of eq 3 for the expanded analyte list under cold-water field conditions; the use of this correction for significantly lower temperature than laboratory conditions had not yet been performed for PFAS analytes. Salinity was found to not affect DGT sampling performance. This study also improves the DGT sampler ability by greatly increasing the sampling surface area (and thus *R*_s_) compared to those of Fang et al. and Wang et al. while delivering greater accuracy in water concentration prediction than the similarly sized DGT sampler Urik and Vrana introduced.^24–26^ In that study, 24 of 41 PFAS detections in the field were within ±100% RPD of grab samples when analyzed similarly to this study, as opposed to the 109 of 117 total found here for two field deployments. The improvements resulted in a DGT passive sampler that accurately, quickly, and consistently measured PFAS in the laboratory (Figure 3) and had good extraction efficiencies and low uncertainty. The results also further support the use of DGT passive samplers which lack an outer membrane, reducing the possibility of PFAS adsorption at the sampler membrane. In the field, the DGT sampler measured PFAS consistently, though underpredicted compared to grab samples (by ∼23% and ∼11% on average in field demonstrations 1 and 2, Figures S4 and S5) either due to an additional environmental factor or grab samples reflecting particle-bound PFAS in addition to the truly dissolved concentration. In both cases, the DGT passive sampler more accurately predicted PFAS water concentration than the MPT passive sampler, and the average was well within the factor of 2 measurement threshold desired for accurate passive sampling. This design of DGT passive sampler along with modeled diffusion-based sampling rates is promising for future field applications and additional development studies. Future studies should focus on better constraining the effects of environmental variables on the DGT passive sampler and improving the FASA performance. Field deployments should track the conditions in which DGT samplers are deployed and evaluate the performance under differing conditions. Though a minimal issue in this study, other preliminary field work has shown our redesigned sampler to be fragile under rough water conditions. Future study should also focus on sampling setups that improve sampler survival in more vigorous environments (such as Image S3). Additionally, for sites where high concentrations are expected, DGT samplers should be deployed for shorter periods to limit competition between PFAS on the adsorbent (and also to avoid exceeding mass-load thresholds determined in this study, potentially leaving the linear uptake regime). This also warrants further study of the DGT/WAX adsorbent capacity. Nonetheless, DGT passive sampling is currently a strong candidate for high-precision PFAS passive sampling in freshwater matrices.

## Supplementary Material





## Data Availability

All PFAS Data is available at https://doi.org/10.7910/DVN/V9PKJN.

## Notes

Additional details on MPT passive sampler use, LC-MS/MS analysis, images of DGT passive samplers, and diffusion and uptake experiment figures (PDF)
PFAS stock details, LC-MS/MS target compound information, and numerical experimental results and internal standard recoveries (XLSX)
