# Supplementary material for: Furthering the Capabilities of Diffusive-Gradient Passive Samplers for Per- and Polyfluoroalkyl Substances
Source: Environ Sci Technol. 2025 May 8;59(19):9744–53. doi: 10.1021/acs.est.4c14136 (PMC12228544; doi:10.1021/acs.est.4c14136)

## Supplemental Information

**Furthering the Capabilities of Diffusive-Gradient Passive Samplers for Per- and Polyfluoroalkyl Substances.**

Jarod Snook[1], Jitka Becanova[1], Simon Vojta[1], and Rainer Lohmann[1*]

[1]Univerisity of Rhode Island Graduate School of Oceanography. 215 S Ferry Rd, Narragansett, RI, 02882, United States.

*corresponding author, Rainer Lohmann (rlohmann@uri.edu)

**Number of Pages: 12**

**Number of Tables: 10**

**Number of Figures: 5**

Table of Contents

Image S1. Assembled DGT passive sampler. ……………………………………………S4

Table S1. Details for PFAS compounds used in spiking solutions. ……………………………………See Excel

Image S2. Slice-Stacking Diffusion method set up. ……………………………………………S4

Table S2. Target Compound List for LC-MS/MS Analysis ……………………………………See Excel

Supplemental Text 1. Description of Diffusion models. ……………………………………………S4

Supplemental Text 2. Microporous Polyethylene Tube Passive Sampler Deployment and Extraction. ……………………………………………S5

Supplemental Text 3. LC-MS/MS analysis details. ……………………………………………S5

Table S3. Method Detection Limits for Various Sample Types. ……………………………………See Excel

Table S4. DGT Extraction Efficiency Results. …………………………………… See Excel

Table S5. Internal Standard Recoveries for Field Samples. …………………………………… See Excel

Table S6. Diffusion Coefficient Results for slice-stacking diffusion experiment and models. …………………………………… See Excel

Figure S1. Stack Diffusion Curve Fitting Results. ………………………………………………S6

Figure S2. 5°C Diffusion Experiment Results. ………………………………………………S8

Figure S3. DGT uptake over time in flow-tank experiment with linear fits. ………………………………………………S9

Table S7. Water concentrations determined in flow-tank DGT calibration. ......................................... See Excel

Table S8. Water concentrations determined in flow-tank DGT “challenge” deployment. ......................................... See Excel

Table S9. Water concentrations determined by DGT, MPT, and grab sampling in Field Demonstration 1. ......................................... See Excel

Figure S4. Passive sampler/grab sample measured concentration ratio for each detection in Field Demonstration 1. ......................................................S10

Table S10. Water concentrations determined by DGT, MPT, and grab sampling in Field Demonstration 2. ......................................... See Excel

Figure S5. Passive sampler/grab sample measured concentration ratio for each detection in Field Demonstration 1. .....................................................S11

Image S3. Example of protective cage set up for DGT samplers in challenging water conditions. .....................................................S12


**Image S1**. Assembled DGT passive sampler.

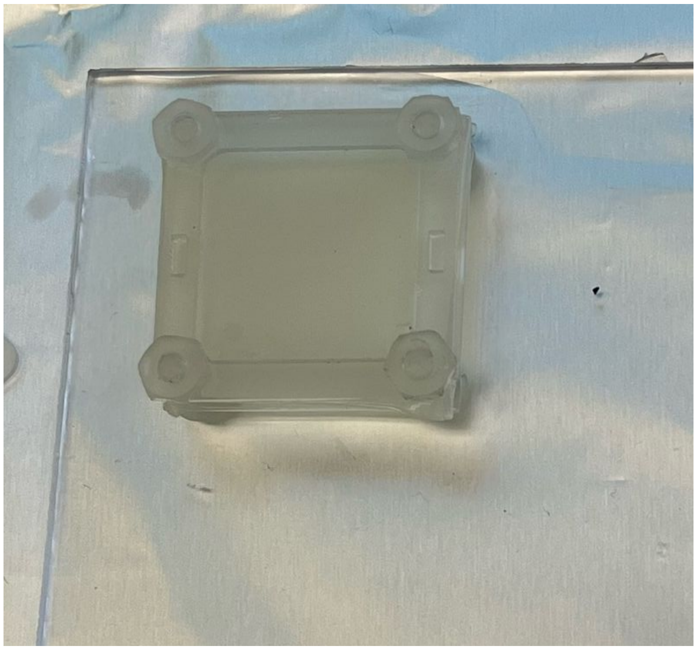

**Image S2**. Stacked agarose sheets for diffusion experiment (left) which are placed in sealed plastic bags in a temperature chamber for 90 minutes.

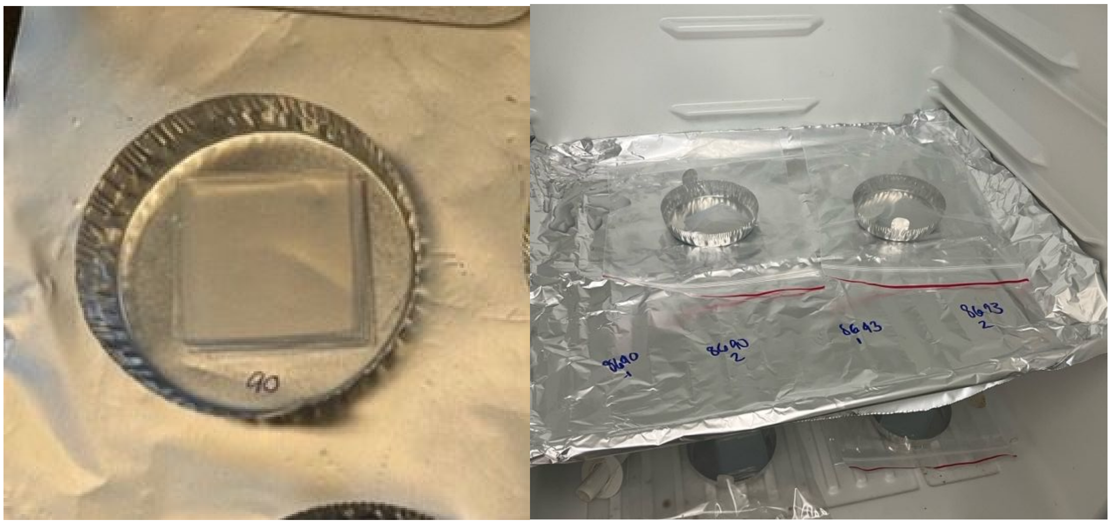


**Supplemental Text 1.**

Diffusion rates were determined by averaging 3 models for diffusion through agarose gel or water based on the individual PFAS molecular weight or volume. The model for diffusion based on Archie's law (Eq. S1) includes parameters adjusting for agarose specific porosity and Archie's law coefficient. The other two models (Eq. S2&S3) describe diffusion through water, but due to the similarity of agarose diffusion (Zhang and Davison, 1999), all three were averaged for a single diffusion coefficient ($D_H$) and

its uncertainty (standard deviation). $D_H$ can then be corrected for a different temperature (Main Text Eq. 3), or converted into sampling rate with Main Text Eq. 6.

$$D_e = \frac{3.3 \times 10^{-5} 0.98^2}{\sqrt[3]{M}} \quad \text{(Eq. S1)}$$

$$D_w = 0.000152 \times V^{-0.64} \quad \text{(Eq. S2)}$$

$$D_w = 0.00007 \times M^{-0.45} \quad \text{(Eq. S3)}$$

M is PFAS molecular weight and V is molecular volume. Eq. 1 comes from Challis et al. 2016, and Eq. 2&3 are from Schwarzenbach et al. 2016.

**Supplemental Text 2.** Microporous Polyethylene Tube Passive Sampler Deployment and Analysis

The MPT passive samplers were handled, in general, following Gardiner et al. 2019, and Dunn et al. 2024. Prepared PE tubes (7cm length microporous polyethylene cylinders containing 600 mg Oasis HLB adsorbent) were cleaned and conditioned by immersion in 3% ammonium hydroxide in LC-MS grade methanol, then 100% LC-MS grade methanol for 24 hours each. Conditioned samplers were stored in LC-MS grade water until deployment. Samplers were deployed alongside DGT for the same deployment lengths via identical rope and zip-tie set up. Upon recovery, MPT samplers were centrifuged repeatedly at 4000 RPM until dry, spiked with internal standard solution, then extracted with methanol in a 2 x 24-hour shaker table extraction process. Methanol extracts were then treated the same as all others in the study with evaporation, recovery standard addition, and LC-MS/MS analysis.

Results from MPT samplers were converted to water concentrations also via sampling rates—sampling rates for the Duluth, MN field study were from Dunn et al. 2024, and MPT sampling rates for the Maine field study were adjusted for temperature using the model described in Dunn et al. 2023. Mathematical conversion to water concentration with sampling rates as well as all blank, MDL, and recovery standard calculations were the same as described for DGT passive samplers.

**Supplemental Text 3.** LC-MS/MS analysis
The instrumental analysis was performed using a SCIEX ExionLC AC UHPLC system coupled to a SCIEX X500R quadrupole time-of-flight tandem mass spectrometer (QTOF MSMS). A Phenomenex Gemini 3um C18 110Å 50x2mm LC analytical column preceded with a Phenomenex SecurityGuard cartridge was used for the analyte separation. Another Phenomenex Gemini 5um C18 110 Å 50x4.6mm LC analytical column was used to delay the PFAS instrumental contribution. The aqueous mobile

phase (MPA) was 10mM ammonium acetate in water, and the organic mobile phase (MPB) was 10mM ammonium acetate in methanol. LC parameters were set to: flow 0.3mL/min, injection 20uL, column oven 45°C. Solvent gradient of MPB gradually increased from 40% to 80% (1 to 5.5 min), 80% to 100% (5.5 to 7 min), then hold for one minute and finally drop to 40% (8 to 8.5 min) and hold for another 6.5 minutes. For the quantification of the target analytes, a HRMSMS (MRM HR) method was used. Negative mode ESI with the following parameters was used: curtain Gas at 30 psi, ion source gas 1 at 40 psi, ion source gas 2 at 60 psi, temperature 450°C.

**Figure S1**. Slice-stacking diffusion experiment results and curve fits. Each of the seven sheets were plotted as distance (from the centroid of the spiked sheet), and mass of a given PFAS over distance was fit with Eq. 1.

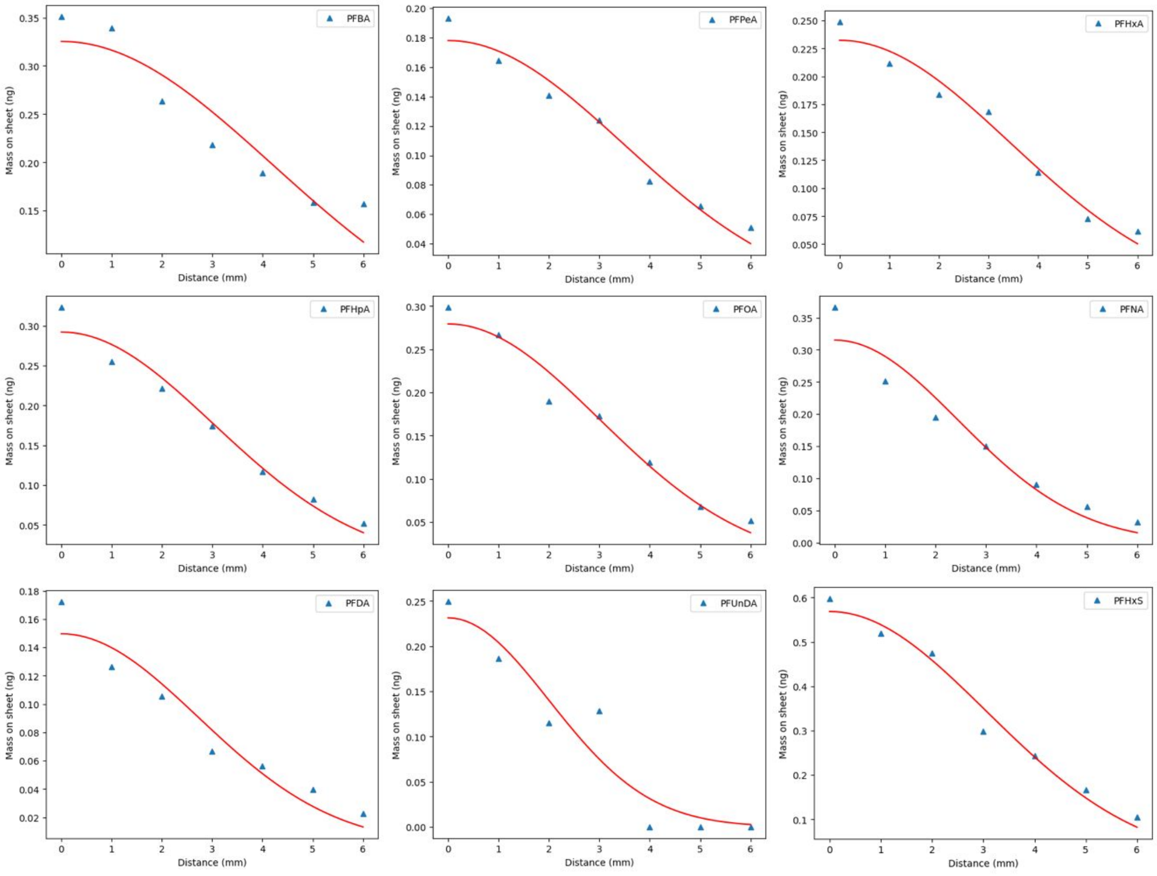

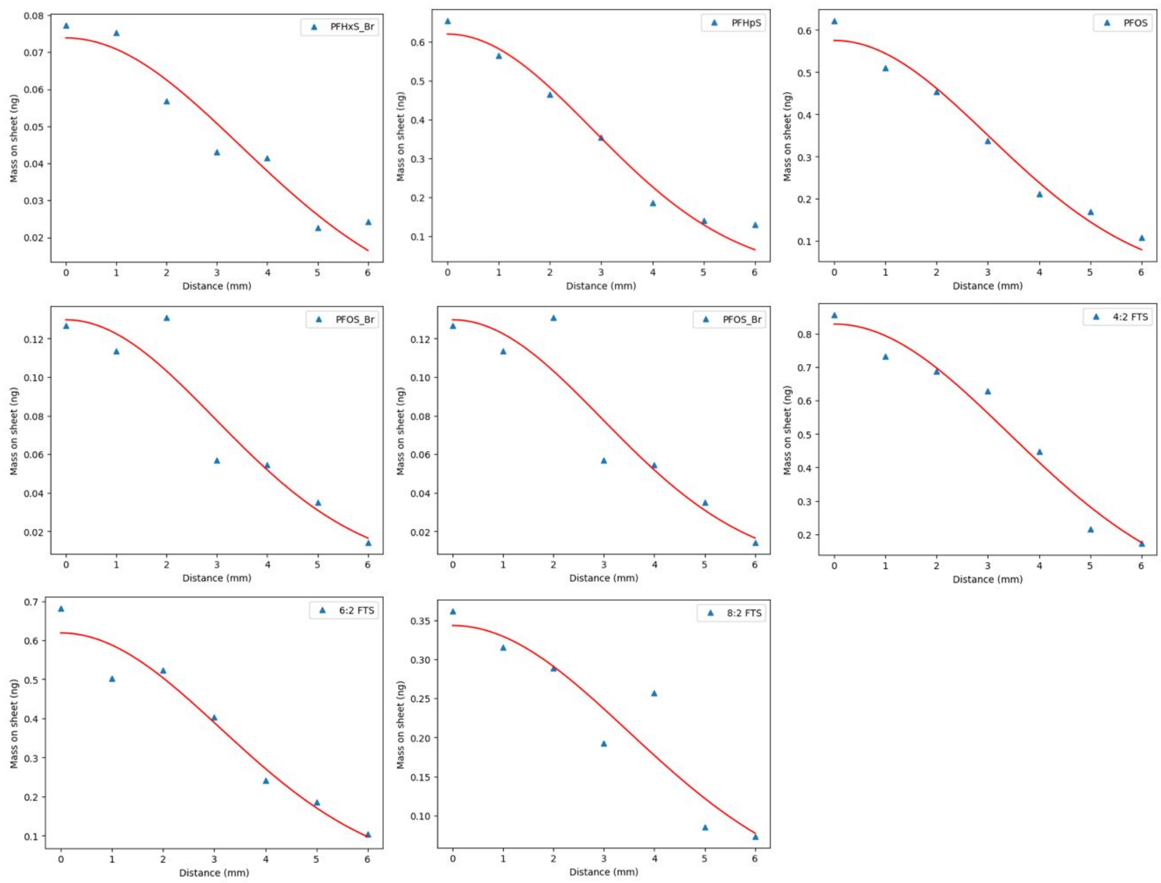
PFHxS_Br
PFHpS
PFOS
PFOS_Br
PFOS_Br
4:2 FTS
6:2 FTS
8:2 FTS
Mass on sheet (ng)
Distance (mm)

**Figure S2**. 5°C Diffusion Experiment Results compared with temperature-adjusted modeled $D_H$.

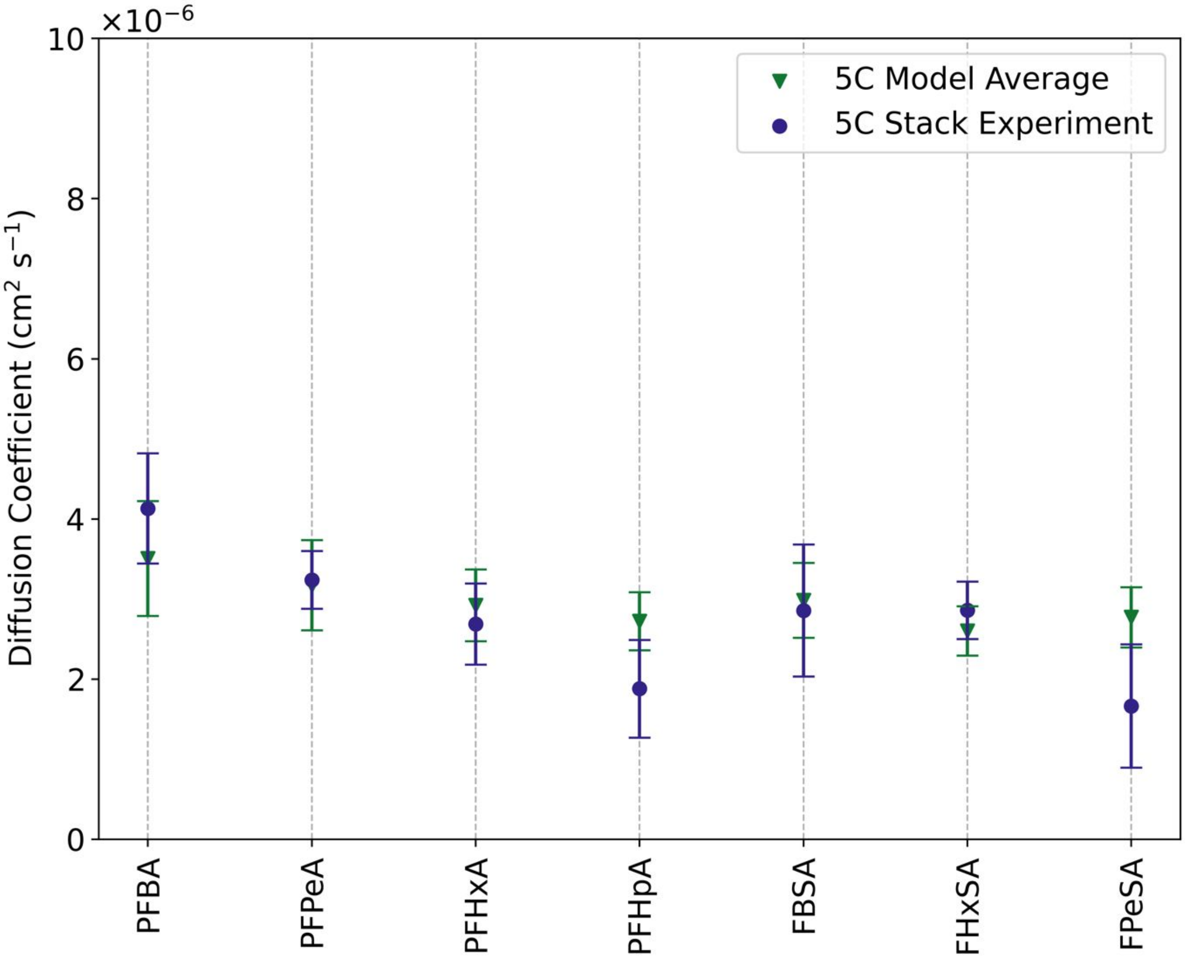

**Figure S3**. DGT uptake over time of each PFAS in flow-tank experiment with linear fit. The ratio of PFAS mass on the DGT sampler (M) to water concentration as measured by grab-samples (Cw) was plotted over time. The linear fit (y-intercept = 0) slope is the experimental sampling rate, as per main text Eq. 4. Analyzing data in this fashion allows multiple experiments, with potentially different Cw values, to be used together for $R_s$ determination.

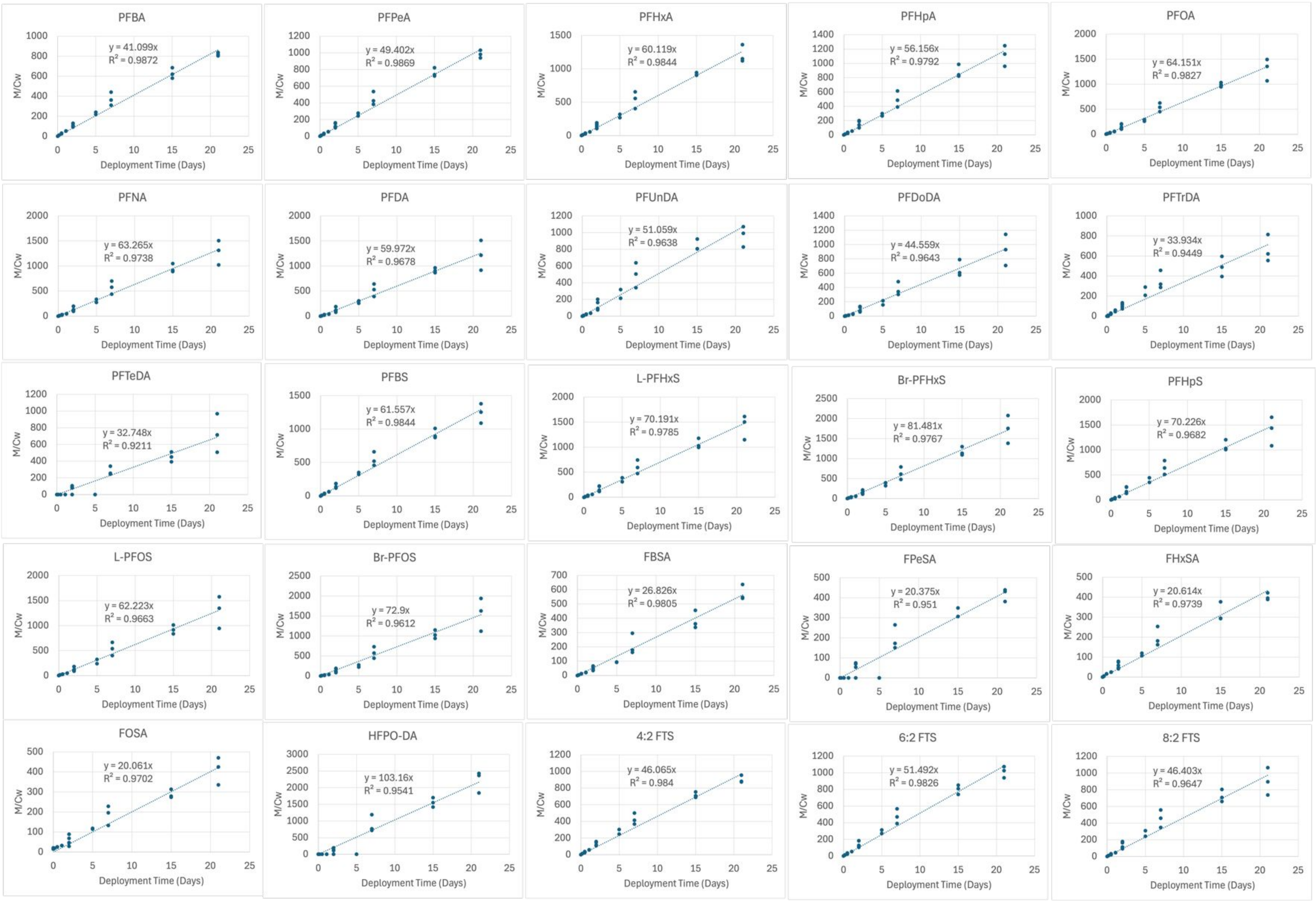

Figure S4. Ratio of passive sampler to grab sample measured PFAS concentration for each detection in Field Demonstration 1. Uncertainty is reflective of both error in grab sample replicate measurements and passive sampler determined concentrations.

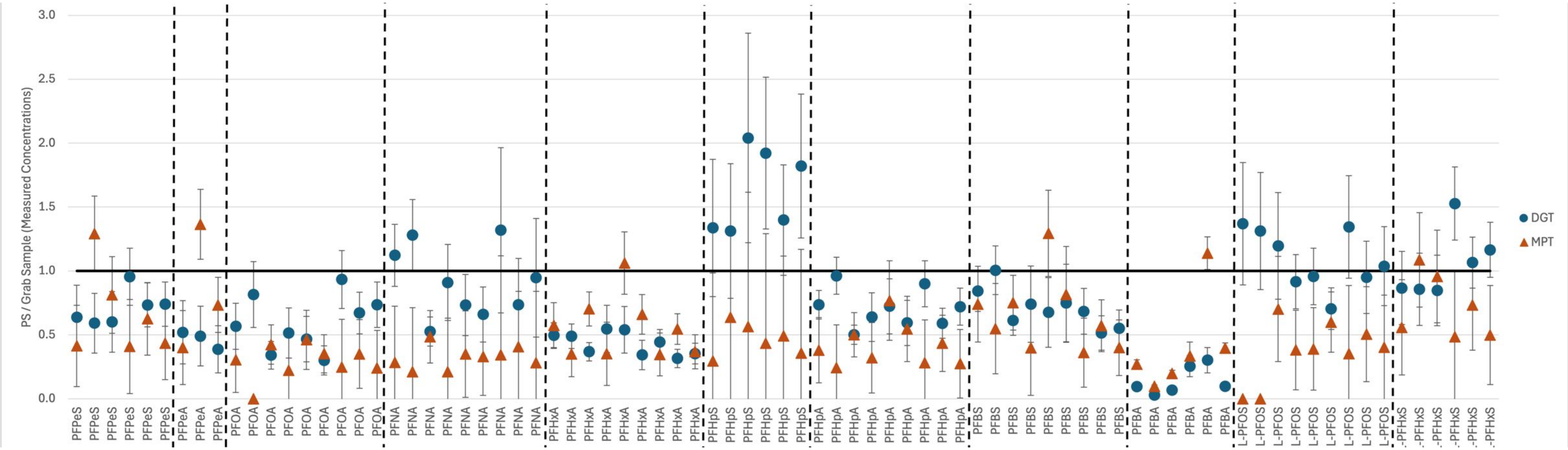

Figure S5. Ratio of passive sampler to grab sample measured PFAS concentration for each detection in Field Demonstration 2. Uncertainty is reflective of both error in grab sample replicate measurements and passive sampler determined concentrations.

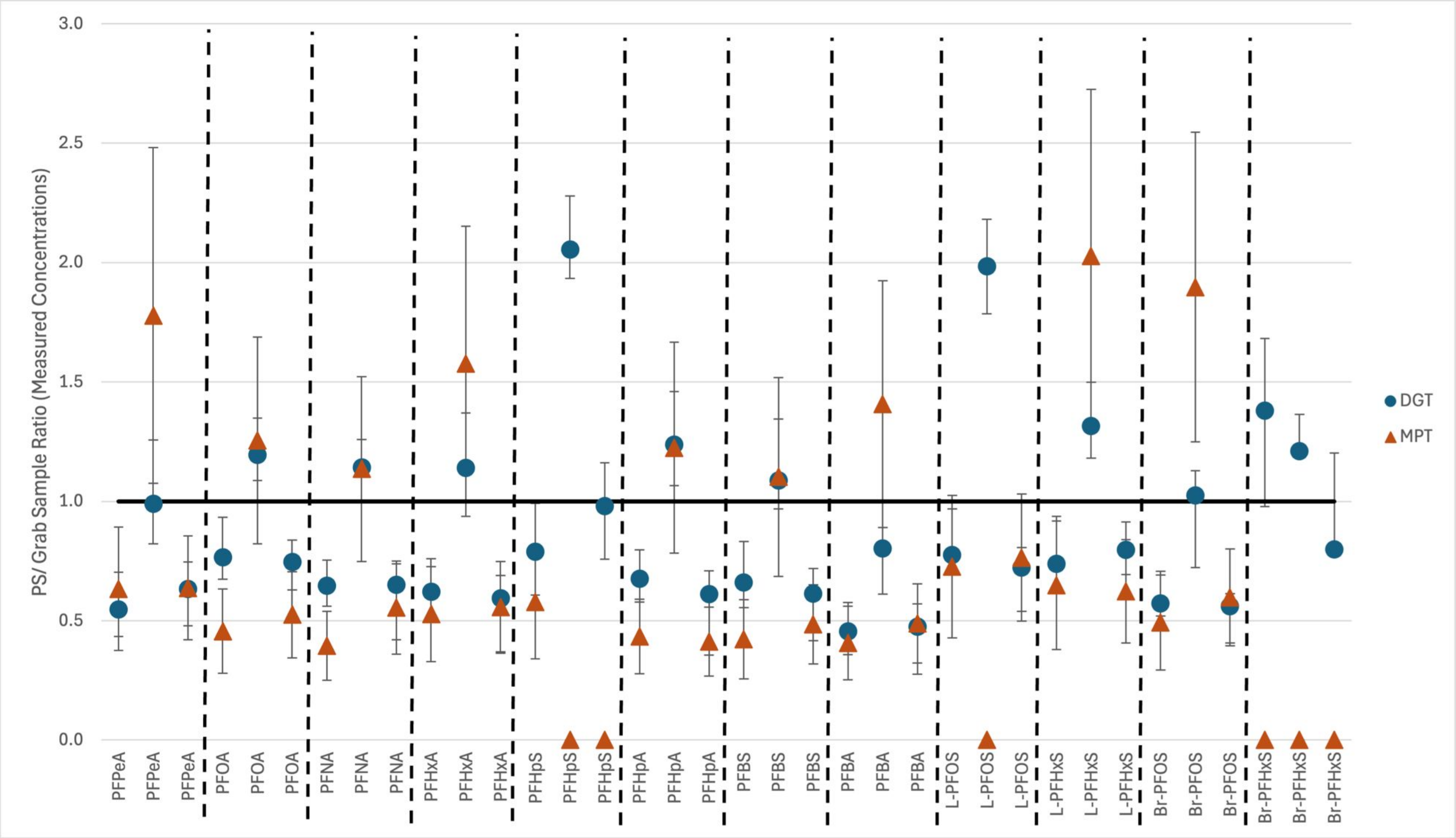

Image S3. Example of DGT sampler protective cage set-up for better survivability in harsh water environments (post-deployment with intact samplers removed).

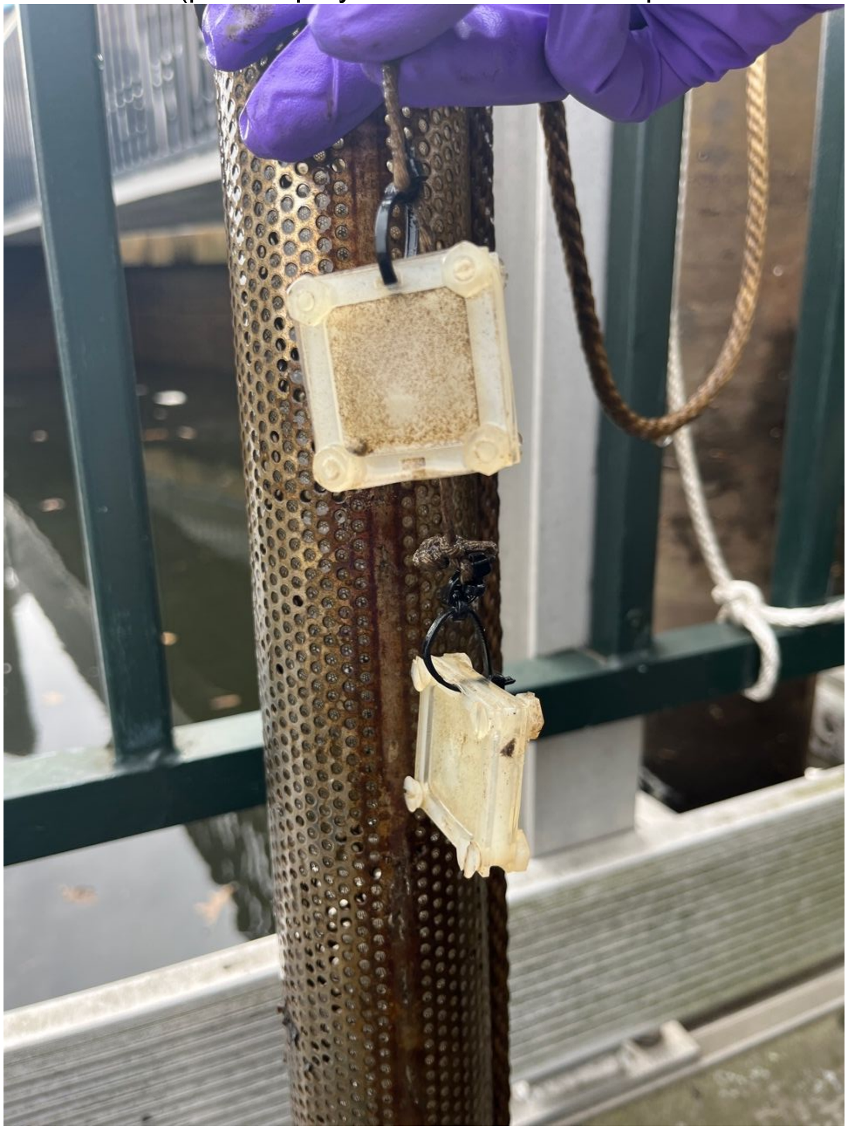

Supplement: Supplementary file 1 [file es4c14136_si_001.pdf]
